# Assessing the Quality of YouTube Videos on Adhesive Capsulitis

**DOI:** 10.7759/cureus.27406

**Published:** 2022-07-28

**Authors:** Kevin Tang, Umair Azhar, Mustufa Babar, Atif Ahmed, Aaron Oh, Wesley Day, Hussein Harb, Ferdinand J Chan

**Affiliations:** 1 Department of Orthopaedic Surgery, Albert Einstein College of Medicine, The Bronx, USA; 2 Radiology, Nova Southeastern University Dr. Kiran C. Patel College of Osteopathic Medicine, Davie, USA; 3 Department of Orthopaedic Surgery, Ross University School of Medicine, Roseau, DMA; 4 Department of Orthopaedic Surgery, Montefiore Medical Center, The Bronx, USA

**Keywords:** quality, social media, patient education, videos, youtube, frozen shoulder, adhesive capsulitis

## Abstract

Introduction

YouTube is the most popular video-based source of information on the Internet. It is accessed by over 1 billion users, which approximates to almost one-third of all Internet users. Orthopaedic video content published on YouTube is not screened and does not go through an editorial process, and most videos do not have information about authorship or appropriate references. Users who do not have the knowledge to assess the accuracy and reliability of the source may be misinformed about their medical condition. Previous studies have evaluated the quality of YouTube content for information in orthopaedics such as meniscus,kyphosis, and anterior cruciate ligament (ACL), but the quality of frozen shoulder videos on YouTube has not been investigated. The purpose of this study is to evaluate the quality and educational value of YouTube videos concerning adhesive capsulitis.

Methods

A YouTube search was performed using the term "frozen shoulder." Videos were excluded if they had no audio, were in a language other than English, or were longer than 10 minutes. A total of 70 videos were screened, and the first 50 videos that met the inclusion criteria were evaluated by three observers. Six video characteristics were extracted, and videos were categorized by source and content. Quality and educational value were assessed using the DISCERN (score range, 0-5), Global Quality score (GQS; score range, 0-4), and a Frozen Shoulder-Specific Score (FSSS; score range, 0-16).

Results

The mean video duration was 242.46 ± 164.32 seconds. The mean number of views was 137,494 ± 262,756 and the total view count across 50 videos was 6,874,706. The mean DISCERN, GQS, and FSSS scores were 2.72 ± 0.85, 2.37 ± 0.895, and 4.42 ± 3.15, respectively. The video sources were primarily from non-physician healthcare professionals (32%), and most of the video content was focused on disease-specific information (50%). Significant between-group effects were observed for the DISCERN score and video source (P = .005), with videos from academic sources having the highest mean DISCERN score. DISCERN scores also differed significantly based on video content (P = .007), with disease content having the highest DISCERN score. Both GQS and FSSS scores differed significantly based on video content (both P < .001) but did not differ significantly based on the video source.

Conclusions

Information about frozen shoulder on YouTube is low quality and has limited educational value. Thus, providers for orthopaedic conditions should warn their patients and provide better alternatives for education.

## Introduction

Today, the easiest way to access quick and broad medical information is to use the internet. A recent estimate showed that there are 4.66 billion active internet users worldwide, which is 59.5% of the global population [1]. With the usage of the Internet increasing by 1,256% from the year 2000 to 2020, it is likely that the use of the Internet by both orthopaedic patients and orthopaedic surgeons to obtain disease-specific information will continue to grow. In their study of 1296 orthopaedic outpatients in 2017, Burrus et al. demonstrated that 84.9% of patients had access to the internet, and 64.7% of those with internet access reported using the internet for obtaining orthopaedic information [2]. However, the reliability and accuracy of orthopaedic information on the internet has been debated in the literature [3-7].

YouTube is the most popular video-based source of information on the Internet. It is accessed by over one billion users, which approximates to almost one-third of all internet users [8]. Orthopaedic video content published on YouTube is not screened and does not go through an editorial process, and most videos do not have information about authorship or appropriate references. Users who do not have the knowledge to assess the accuracy and reliability of the source may be misinformed about their medical condition. Therefore, it is essential for orthopaedic surgeons to be aware of and evaluate YouTube sources to help educate patients and guide them through their disease management process.

Adhesive capsulitis, also known as "frozen shoulder," is one of the most common disorders in sports medicine, characterized by pain and stiffness of the shoulder. It is a progressive disorder that restricts both active and passive range of motion of the shoulder, and it is more common in people between the ages of 40 and 60 [9]. Previous studies have evaluated the quality of YouTube content for information in orthopaedics such as meniscus [3], kyphosis [4], and anterior cruciate ligament (ACL) [5], but the quality of frozen shoulder videos on YouTube has not been investigated. In this study, we aim to evaluate the quality and accuracy of the educational information on frozen shoulders shared on YouTube.

## Materials and methods

Search strategy

**Figure 1:**
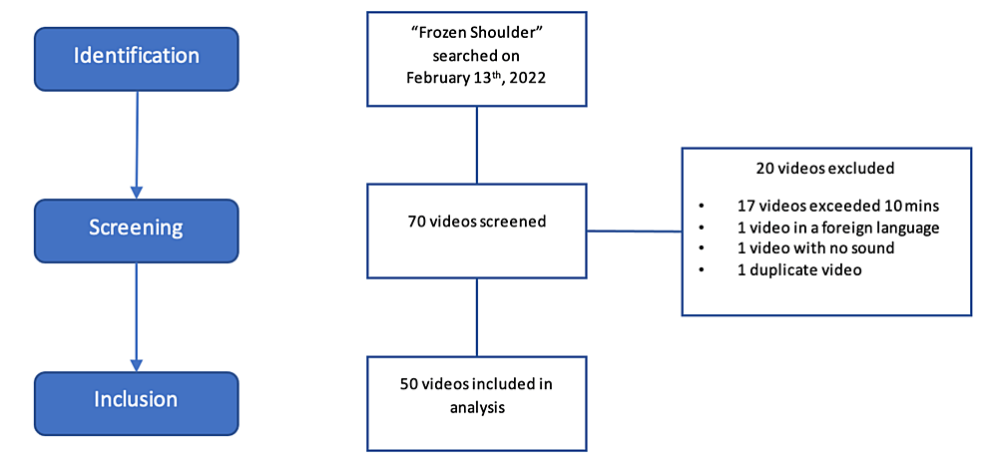
Video Inclusion Flow

The term "frozen shoulder" was searched on YouTube on February 13, 2022, in a cookie-free, cache-cleared, private browsing setting on Google Chrome. The search results were based on YouTube's default settings of proposed relevance. Videos were included if they were relevant to the frozen shoulder. Videos were excluded if they had no accompanying audio, narration, and/or text, were in a language other than English, or were longer than ten minutes in length since viewer engagement significantly reduces in lengthier videos [10]. The first 50 videos that met the inclusion criteria were included (Figure 1).

Video parameters and evaluations

Video parameters including video length, number of views, video source/uploader, duration on the platform, and number of likes were extracted. The view ratio was calculated by dividing the number of views by the duration on the platform. Videos were classified into one of seven source categories: academic, commercial, medical source, non-physician healthcare professional, patient, physician, or trainer. Videos were classified into one of six content categories: patient experience, disease-specific information, exercise training, surgical technique/approach, nonsurgical management, or advertisement.

Three instruments were used to assess the quality of the information in the videos: DISCERN, Global Quality Score (GQS), and Frozen Shoulder-Specific Score (FSSS). DISCERN contains 15 questions, each scored on a 5-point scale, and is used to evaluate the quality of consumer health information regarding treatment options [10]. In this study, a modified five-question, 5-point scale DISCERN score was used to quantify the reliability of the information in the videos (Table 1). GQS provides a non-specific assessment of educational value through a 5-point scale (Table 2). We generated FSSS to better assess the educational content of information on frozen shoulder. The FSSS was composed of 16 items derived from guidelines published by the American Academy of Orthopaedic Surgeons [11] and evaluated information on common patient presentations and symptoms, anatomy, diagnosis and evaluation, treatment, and postoperative course and expectations (Table 3). For each item present, one point was assigned for a maximum score of 16. For all three instruments used in this study, higher scores indicated a better overall quality of information.

**Table 1:** DISCERN Criteria

Reliability of information (1 point for every Yes, 0 points for No)
Are the aims clear and achieved?
Are reliable sources of information used? (i.e., publication cited, speaker is board-certified physician)
Is the information presented balanced and unbiased?
Are additional sources of information listed for patient reference?
Are areas of uncertainty mentioned?

**Table 2:** GQS Criteria GQS: Global Quality Score

Grade	Description of quality
1	Poor quality and unlikely to be of use for patient education
2	Poor quality and of limited use to patients because some information is present
3	Suboptimal quality and flow; somewhat useful to patients; important topics are missing; some information is present
4	Good quality and flow; useful to patients because most important topics are covered
5	Excellent quality and flow; highly useful to patients

**Table 3:** FSSS Criteria for Video Content FSSS: Frozen Shoulder-Specific score; MRI: magnetic resonance imaging; PT: physical therapy; NSAIDs: non-steroidal anti-inflammatory drugs

Patient presentation
Describes symptoms
Describes patient population
Information about frozen shoulder
Describes anatomy and/or function of the shoulder
Describes frozen shoulder pathophysiology
Describes mechanism of injury
Diagnosis and evaluation
Describes physical examination and findings
Mentions that external rotation deficit is most common
Discusses that X-rays needed to evaluate for osteoarthritis, dislocation, or surgical hardware
Discusses that MRI is not necessary for diagnosis but can evaluate for other pathology
Treatment
Discusses conservative treatment (PT, NSAIDS, and intra-articular steroid injections) as first line treatments for 3-6 months
Mentions diagnostic arthroscopy and other pathologies that can be addressed concomitantly (rotator cuff tears, biceps pathologies, etc.)
Describes manipulation under anesthesia and arthroscopic lysis of adhesions
Postoperative course
Describes complications and outcomes
Mentions the need for physical therapy after operation
Outlines return-to-function timeline

Three authors collected data and evaluated the videos. If videos fit into more than one content category, they are classified into the category that captures the primary focus of the video content. Disagreements about classification were settled by discussion and consensus.

Statistical analysis

Video characteristics, video reliability, and video quality scores were quantified using descriptive statistics. One-way analysis of variance (ANOVA) was used for parametric data and its non-parametric equivalent, Kruskal-Wallis, was used for nonparametric data to determine whether FSSS, GQS, and DISCERN scores differed based on either video source or video content. If the Kruskal-Wallis test was significant, then a Dunn Test was performed to determine which exact groups within the video source and video content differed significantly. Interobserver reliability defined by interobserver correlation coefficient scores (ICC) was 0.68 (95% confidence interval [95% CI], 0.49-0.81) for DISCERN, 0.94 (95% CI, 0.91-0.97) for FSSS, and 0.88 (95% CI, 0.81-0.93) for GQS. All statistical tests were performed using R Programming (version 4.1.2). P < .05 was considered to indicate statistical significance.

## Results

A total of 70 videos were screened before 50 met inclusion. The mean video duration was 242.46 ± 164.32 seconds. The mean number of views was 137,494 ± 262,756 and the total view count was 6,874,706. The mean view ratio was 95.42 ± 145.87 and the mean number of days since uploaded was 1346.46 ± 1034.67. The mean number of likes was 1264.98 ± 2912.41. The mean DISCERN, GQS, and FSSS scores were 2.72 ± 0.85, 2.37 ± 0.895, and 4.42 ± 3.15, respectively. Videos were primarily categorized into disease-specific information (50%), followed by nonsurgical management (40%), and then patient experience (10%). The video sources were primarily from non-physician healthcare professionals (32%) or physicians (30%).

The DISCERN scores differed significantly based on video source (P = .005), with videos from academic, commercial, and physician sources having the highest DISCERN scores (commercial: 4.33; academic: 3.78; physician: 3.16). DISCERN scores also differed significantly based on video content (P = .007), with disease content and nonsurgical disease management having the highest DISCERN scores (disease-specific information: 3.12; non-surgical: 2.43). The FSSS scores differed significantly based on video content (P < .001), with disease and patient experience content having the highest FSSS scores, but did not differ significantly based on video source (P = .09). The GQS score differed significantly based on video content (P < .001), with disease and patient experience content having the highest GQS scores but did not differ significantly based on video source (P = .057) (Table 4).

**Table 4:** Mean DISCERN, GQS, and FSSS Scores Based on Video Content and Video Source Categories NOTE: Data are presented as mean (SD). For video content, between group effects showed P = .001 for DISCERN and P < .001 for both GQS and FSSS. For video source, between group effects showed P = .0054 for the DISCERN score, P = .05 for GQS, and P = .09 for FSSS. GQS: Global Quality Score; FSSS: Frozen Shoulder-Specific Score.

Group variable	N (%)	DISCERN	GQS	FSSS
Video content				
Patient experience	5 (10)	1.93 (1.04)	2.13 (0.77)	4.27 (2.58)
Non-surgical management	20 (40)	2.43 (0.59)	1.73 (0.53)	1.93 (1.61)
Disease-specific information	25 (50)	3.12 (0.81)	2.94 (0.79)	6.44 (2.78)
Video source				
Academic	3 (6)	3.78 (0.69)	3.22 (1.02)	7.22 (3.91)
Commercial	1 (2)	4.33 (0)	2.50 (0)	5.67 (0)
Medical	12 (24)	2.36 (0.36)	2.22 (0.69)	3.47 (2.59)
Non-physician healthcare professional	16 (32)	2.48 (0.82)	1.88 (0.77)	2.90 (2.42)
Patient	2 (4)	1.83 (1.18)	2.17 (0.71)	4.83 (3.06)
Physician	15 (30)	3.16 (0.74)	2.91 (0.91)	6.16 (3.45)
Trainer	1 (2)	1.67 (0)	2.00 (0)	3.67 (0)
Total	50 (100)	2.73 (0.85)	2.38 (0.90)	4.42 (3.15)

Among video content, the Dunn test showed a significant difference in DISCERN score between patient experience and disease content (P = .029) and between nonsurgical management and disease content (P = .006). The Dunn test showed significant differences between nonsurgical management and disease content for FSSS scores (P < .001) and GQS scores (P < .001). Among video sources, the Dunn test showed significant differences in DISCERN scores for academic versus medical sources (P = .009), academic versus patient (P = .031), academic versus trainer (P = .019) and commercial versus trainer (P = .029).

Three separate multivariate linear regression models were constructed to determine the influence of video characteristics on the DISCERN, GQS, and FSSS scores. Significant positive independent predictors of GQS included number of days uploaded (β = .0003, P = .031) and the view ratio (β = .0044, P = .024). GQS had no significant negative independent predictors. Significant positive independent of FSSS included the number of days uploaded (β = .0015, P = .009) and the view ratio (β = .015, P = .022). FSSS had no significant negative independent predictors. DISCERN did not have any significant positive or negative independent predictors from video characteristics.

## Discussion

The findings of this study were as follows: (1) videos concerning frozen shoulders are highly watched by YouTube users with a total view count of 6,874,706 across the 50 videos; (2) the study had a mean DISCERN of 2.73, GQS of 2.38, and FSSS of 4.42 suggesting low-content reliability; (3) most frozen shoulder video content is disease-specific information and the most uploads are from non-physician healthcare provider sources; (4) DISCERN scores differed significantly based on video content and video source while GQS and FSSS scores differed significantly based on video content, but not video source; (5) video characteristics such as the number of days uploaded and the view ratio are significant positive predictors of GQS and FSSS scores; meanwhile, DISCERN had no individual predictors.

Due to the ease of access to information, patients are increasingly using YouTube as a source of information [12]. Our study showed that the total number of views across 50 frozen shoulder videos was 6,874,706, which is similar to what Erdem and Karaca found for 50 "kyphosis" videos (6,582,221) but significantly less than what Kunze et al. found for 50 "meniscus" videos (14,141,285) [3,4]. Information on the disease processes, therapeutic management, or general information on the frozen shoulder is highly sought after given the high number of views that frozen shoulder videos accumulate on YouTube.

This study had a mean DISCERN of 2.73, the GQS was 2.38, and the FSSS was 4.42. These results suggest that the quality of information regarding frozen shoulders obtained from YouTube is poor, which is consistent with other YouTube video studies done in orthopaedics. When evaluating YouTube videos regarding kyphosis [4], Erdem and Karaca determined that the mean JAMA score, GQS, and kyphosis-specific score (maximum of 32 points) were 1.34, 1.68, and 3.02, respectively. In an analysis of disc herniation videos on YouTube, Gokcen and Gumussuyu found that the mean JAMA score and DISCERN score (a comprehensive version of the modified DISCERN score we used) were 1.7 and 30.8, respectively [13]. Cassidy et al. determined that the mean JAMA score and ACL-specific score (maximum of 25 points) were 2.4 and 5.5, respectively [5]. From the collective evaluation of these studies, it can be concluded that the quality of orthopaedics-related video content is poor on YouTube.

In addition, while academic sources had the highest scores for DISCERN (3.78), GQS (3.22), and FSSS (7.22), all well above the mean for each respective score, academic sources only accounted for 6%, or 3 out of the 50 videos that were analyzed. In contrast, 16 out of 50 videos, or 32% of the videos, were uploaded by non-physician healthcare professionals, but these videos had lower DISCERN (2.48), GQS (1.88), and FSSS (2.90) scores than the mean of the scores across the 50 videos. Given the low number of uploads by academic sources and high uploads but low-quality information from non-physician healthcare providers, YouTube users have a lower chance of coming across reputable information concerning frozen shoulder. Regarding content categories, disease-specific information had the highest DISCERN (3.12), GQS (2.94), and FSSS (6.44) scores, all above the mean, and accounted for 25% or 50% of the videos analyzed. Videos describing non-surgical management accounted for 40% of the videos; however, they had relatively low DISCERN (2.43), GQS (1.73), and FSSS (1.93) scores compared to the mean. Further analysis from this study showed that DISCERN scores differed significantly based on video content and video source, with disease-specific information and academic sources having the highest DISCERN scores. GQS and FSSS scores only differed significantly based on video content, with disease-specific information having the highest scores for both GQS and FSSS.

Regression analysis revealed that the number of days uploaded and the view ratio were significant positive predictors of both DISCERN and GQS. This implies that more recent videos have had less reliable content regarding frozen shoulders. This could be due to the significant growth of YouTube as a platform for content creators. The quality of videos has decreased with more content being created by non-physician content creators. It is also interesting to note that FSSS had no significant predictors, and the number of views and likes was not a significant predictor for any score. This demonstrates that the quality of information for frozen shoulder videos cannot be predicted by the popularity of the video.

There are a few limitations inherent to our study. Our study utilized DISCERN, GQS, and FSSS, which are highly regarded but not validated assessment tools. Despite this, numerous other studies that evaluated the reliability and accuracy of internet content for orthopaedic diseases have used these evaluation methods to provide reproducible measures [3-5,13]. Having observers view and rate videos may limit their external validity as they may not represent how patients or physicians view videos. Nevertheless, our use of three trained screeners resulted in excellent agreement and high interobserver reliability, with interobserver correlation coefficient scores of 0.68, 0.94, and 0.88 for DISCERN, FSSS, and GQS, respectively. The first 50 videos that met inclusion criteria were used for the purpose of this study. Though this number may be relatively low, studies have shown that most people do not look beyond the first two search pages [14]. Additionally, our study excluded videos that were longer than 10 minutes, which may have removed videos that had more time to go in depth and therefore may have obtained higher scores. However, the objective of this study was to emulate and analyze results that an average user may encounter when visiting YouTube rather than analyzing all the information available on the platform. Another limitation was the use of "frozen shoulder" as the search term rather than "adhesive capsulitis," which could yield different video results. Despite this, patients may be more familiar with the common term and therefore more likely to search using "frozen shoulder." YouTube is a large platform with a constant flux of video uploads and removals. The dynamic nature of the platform can lead to variations in the videos delivered based on a search algorithm that is influenced by a user’s search history and internet protocol (IP) address. It was not possible to adjust or standardize for this unknown. While YouTube is the most popular video-sharing platform globally [15], it is not the only source of video content. Other video-sharing platforms such as Facebook Watch, Instagram Reels, Vimeo, and TikTok may also include relevant videos but are not included in this study.

## Conclusions

YouTube is becoming an increasingly powerful tool to obtain many forms of information. Therefore, it is important for healthcare providers to be aware of its use and potential for misuse, as there is currently no peer-reviewed system in place for YouTube videos. Thus, providers should engage with their patients by asking them if they have used online resources to understand their condition and should help patients better utilize these resources. While videos on YouTube concerning frozen shoulder are highly viewed, they are, on average, of low quality and low educational value. It may be prudent for those who utilize YouTube to learn more about the risk factors, pathophysiology, symptoms, and medical and surgical management associated with frozen shoulder by complementing their education with other sources that are peer-reviewed and scientifically supported. If providers were able to properly address these resources with patients and inform them about their use, they may be able to help patients find a sense of control in their care for frozen shoulders.
